# Alternative Splicing in Plant Immunity

**DOI:** 10.3390/ijms150610424

**Published:** 2014-06-10

**Authors:** Shengming Yang, Fang Tang, Hongyan Zhu

**Affiliations:** Department of Plant and Soil Sciences, University of Kentucky, Lexington, KY 40546, USA; E-Mail: tangfang0401@uky.edu

**Keywords:** alternative splicing, disease resistance, TIR-NBS-LRR, CC-NBS-LRR, RNA processing, post-transcriptional regulation

## Abstract

Alternative splicing (AS) occurs widely in plants and can provide the main source of transcriptome and proteome diversity in an organism. AS functions in a range of physiological processes, including plant disease resistance, but its biological roles and functional mechanisms remain poorly understood. Many plant disease resistance (*R*) genes undergo AS, and several *R* genes require alternatively spliced transcripts to produce R proteins that can specifically recognize pathogen invasion. In the finely-tuned process of R protein activation, the truncated isoforms generated by AS may participate in plant disease resistance either by suppressing the negative regulation of initiation of immunity, or by directly engaging in effector-triggered signaling. Although emerging research has shown the functional significance of AS in plant biotic stress responses, many aspects of this topic remain to be understood. Several interesting issues surrounding the AS of *R* genes, especially regarding its functional roles and regulation, will require innovative techniques and additional research to unravel.

## 1. Introduction

Alternative splicing (AS) describes the processing of a single pre-mRNA to produce multiple transcript isoforms [1]. Genome-wide studies have shown that AS is prevalent in eukaryotes and that more than 95% of human multi-exon genes undergo AS [2,3]. One of the most impressive examples of AS is the *Drosophila melanogaster* gene *down syndrome cell adhesion molecule* (*Dscam*), which contains 95 exons, and can generate 38,016 distinct alternative transcript isoforms, a number in excess of the total number of genes (14,500) in the genome [4]. AS appears to serve as the primary source for transcriptome and proteome diversity in many eukaryotes [2,5,6,7]. In plants, analysis of *Arabidopsis* EST/cDNA libraries initially gave rise to estimates of AS rates as low as 1.2% [8]. Subsequently, improved EST coverage led to estimates of 11.6% [9], 21.8% [10], and 30% [11]. More recently, high-throughput sequencing has revealed that about 61% of intron-containing genes in *Arabidopsis* undergo AS [12]. Considering that these data were obtained from plants growing under normal conditions, the actual value for AS frequency is likely to be even higher. Environmental and biotic stresses can induce AS, and novel splicing sites have been identified in studies of AS under stress conditions [13,14,15]. A recent RNA-seq study of *Pseudomonas syringae*-infected *Arabidopsis* indicated that over 90% of the expressed genes (23,385 out of 25,619) underwent AS [15]. Moreover, differential expression of alternative transcript isoforms in different tissues and at different development stages adds another layer of complexity to AS mechanisms and transcriptome annotation [16,17,18,19].

Proteins encoded by AS isoforms can have different activities, tissue distributions, or intracellular localizations [17,20,21,22,23]. Although its biological function is not fully understood in plants, AS is involved in many physiological processes, including defense responses [24,25,26,27]. Plants have evolved sophisticated systems to detect pathogen attacks and trigger innate immunity. Recently, AS has been recognized as a crucial regulatory mechanism in plant defense against pathogen infections [28,29,30,31,32]. This review begins with an overview of disease resistance in plants and then discusses current knowledge about the involvement of AS in plant immunity, as well as the prospects for future research.

## 2. Plant Disease Resistance

Two types of plant immunity operate to restrict pathogen colonization in the host. The first, a basal level of plant defense responses are activated by the pathogen (or microbe)-associated molecular patterns (PAMPs or MAMPs), such as chitin, flagellin, and Elongation Factor-Tu (EF-Tu). The perception of structurally conserved PAMPs by plant transmembrane pattern recognition receptors (PRRs) induces PAMP-triggered immunity (PTI). However, pathogens can suppress PTI with secreted effector proteins. Accordingly, in the second line of defense, the plant deploys resistance (R) proteins to recognize corresponding effector proteins called Avirulence (Avr) proteins, leading to the stronger disease resistance, called effector-triggered immunity (ETI). R proteins recognize Avr proteins either directly or indirectly. Direct R-Avr interaction is exemplified by the direct binding of the *Linum usitatissimum* (flax) L. protein with its cognate effectors [33]. Indirect R-Avr interaction can be explained by the proposed “guard hypothesis” [34]; in this model, R proteins detect pathogens indirectly, by the effects of Avr proteins on other cellular proteins, termed guardees.

The co-evolution or “arms race” between host and pathogen has been extensively studied in the interaction between *Arabidopsis* and pathogenic *P. syringae* expressing EF-Tu. Direct binding of EF-Tu to its receptor EFR induces phosphorylation on the tyrosine residues of EFR, and activates PTI [35]. However, the *P. syringae*-secreted effector HopA1 has phosphatase activity and reduces EFR phosphorylation, thus blocking EF-Tu-triggered PTI [36]. The *Arabidopsis* R protein RPS6 (Resistance to *P. syringae* 6) specifically recognizes HopA1 [37]. The HopA1 target guarded by RPS6 is believed to be EDS1 (Enhanced disease susceptibility 1), a pivotal signal transducer in RPS6-mediated ETI, although EDS1 also functions downstream of pathogen detection [38].

PTI cannot completely inhibit pathogen colonization, but can retard pathogen invasion [39]. By contrast, ETI can be viewed as intensified and long-lasting PTI that includes the development of systemic acquired resistance and rapid, localized programmed cell death known as the hypersensitive response (HR) [40]. A chain of defense responses occur concomitant with the HR, including oxidative burst, accumulation of salicylic acid (SA), expression of pathogenesis-related (PR) genes, and defensin biosynthesis. PTI involves mitogen-activated protein kinase-signaling cascades and the accumulation of reactive oxygen species [41,42], and constitutive activation of PTI in the absence of pathogen results deleterious effects on plant development. As a long-lasting, systemic response, ETI must be fine-tuned to protect the plant from pathogen attack without excessive fitness costs.

### 2.1. R Genes

The majority of cloned *R* genes encode proteins containing a central nucleotide-binding site (NBS) and a *C*-terminal leucine-rich repeat (LRR) region. The NBS region normally consists of three subdomains, NBS, ARC1, and ARC2. The characteristic NBS subdomain includes a binding site for ATP or GTP and is active in initiation of signaling cascades leading to resistance responses [43]. The ARC subdomains (named for their presence in Apaf-1, R proteins, and CED-4) are highly conserved and essential for intramolecular interactions of R proteins [44]. By contrast, the LRR motif confers recognition specificity to the plant defense response [45,46,47,48].

Based on their *N*-terminal structures, members of the NBS-LRR family of *R* genes can be further subdivided into two subfamilies. One subfamily comprises members with a domain homologous to the intracellular signaling domains of the *Drosophila* Toll and mammalian Interleukin (IL)-1 receptor (TIR-NBS-LRR). TIR-NBS-LRR genes are exclusively present in dicot species. Members of this subfamily include tobacco *N*, flax *L6* and *M*, *Arabidopsis RPP1*, *RPP4* and *RPS4*, and *Medicago truncatula RCT1*. Another subfamily is characterized by a putative coiled-coil domain in the *N*-terminal region (CC-NBS-LRR). CC-NBS-LRR genes are widely distributed in both dicots and monocots. Both the CC and TIR domains likely function in interaction with downstream factors in ETI signaling [49]. Although most TIR- and CC-NBS-LRRs lack putative transmembrane domains or organelle-targeting signals and are predicted to be cytosolic, some show dynamic changes in subcellular localization [42,50].

### 2.2. Signaling Components in ETI

In addition to their structural differences, TIR-NBS-LRR and CC-NBS-LRR genes generally function through distinct signaling pathways, requiring either EDS1 or NDR1 (Non-race-specific disease resistance 1), respectively [51]. One exception is the *Arabidopsis HRT* gene that confers resistance to TCV (Turnip crinkle virus). HRT is a CC-NBS-LRR gene but its signaling is dependent on EDS1 [52]. Moreover, a few CC-NBS-LRR genes including *RPP7*, *RPP8*, and *RPP13* can activate defense signaling independent of EDS1 and NDR1 [51,53,54]. Venugopal *et al.* [55] proposed, however, that EDS1 and SA act redundantly to regulate ETI to viral, bacterial, and oomycete pathogens. As such, participation of EDS1 in signaling triggered by CC-NBS-LRR R proteins may be masked by SA, and *vice versa*. In such cases, the requirement for EDS1 would be observed only when disease resistance does not require SA accumulation. PAD4 (Phytoalexin deficient 4) and SGA101 are indispensable for EDS1-required signaling to restrict pathogen growth [56,57]. EDS1, PAD4, SAG101 function independently, as well as in a ternary complex of SAG101-EDS1-PAD4, serving as signal transducers in HRT-mediated resistance to TCV [58]. However, the HR associated with TCV resistance conferred by HRT requires only EDS1, whereas the SA signaling induced by HRT requires only PAD4.

Genetic analysis of *Arabidopsis* mutants defective in systemic acquired resistance led to the isolation of *NPR1* (*Non-expresser of PR genes 1*), which encodes a putative transcription factor regulating *PR* gene expression downstream of SA production [59]. Further investigation of the regulator of *NPR1* in *Arabidopsis* resulted in identification of the gain-of-function mutant *snc1* (*Suppressor of npr1-1*, *constitutive 1*) [60], which exhibits a dwarfed phenotype caused by constitutive activation of defense signaling in the absence of pathogen infection. Based on these mutants it can be concluded that wild type SNC1 suppresses NPR1 and to finely control autoimmune responses. Interestingly, *snc1* encodes a TIR-NBS-LRR R protein, and the *snc1* mutant morphology is restored or suppressed to different extents in a series of *mos* (*Modifier of snc1*) mutants. Thus far, 13 *MOS* genes have been cloned, the gene products of which act in various cellular and molecular processes, including pre-mRNA splicing, nuclear trafficking of serine-arginine rich (SR) proteins and protein modification, which is indicative of a highly complex network for regulation of R protein-mediated ETI [61,62,63,64,65,66,67,68,69,70,71,72].

## 3. AS of *R* Genes

### 3.1. AS of TIR-NBS-LRR Genes

Most TIR-NBS-LRR genes have conserved gene structures in the coding region, which generally contains three or four introns. The first exon encodes the TIR domain, the second exon encodes the NBS domain, and the remaining exons encode the LRR region. AS of TIR-NBS-LRR genes can result from intron retention, selection of alternative exons, or usage of alternative 5' or 3' splicing sites. Alternative isoforms have been reported for many TIR-NBS-LRR genes, such as tobacco *N* [73], flax *L*, and *M* loci [74], *Arabidopsis SNC1*, *RPS4*, *RPS6*, *RPP5*, and *RAC1* [37,75,76,77,78], tomato *Bs4* [79], potato *Y-1* [80], and *M. truncatula RCT1* [81]. The functional consequences of AS events have been characterized for only a few TIR-NBS-LRR *R* genes, including *Arabidopsis RPS4*, tobacco *N*, and *M. truncatula RCT1*.

#### 3.1.1. *Arabidopsis RPS4*

**Figure 1:**
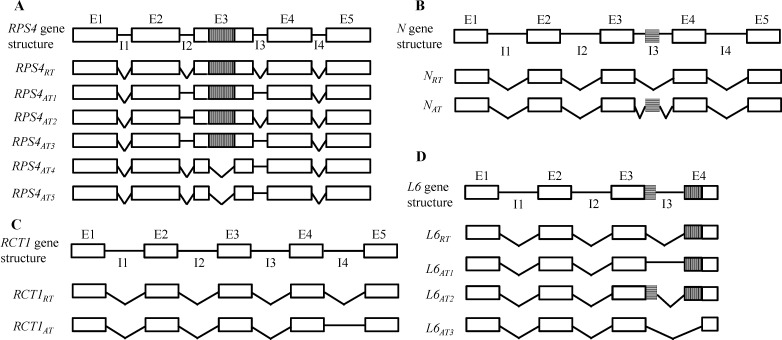
Schematic diagramof gene structure and transcript isoforms for the TIR-NBS-LRR genes *Arabidopsis RPS4* (**A**); tobacco *N* (**B**); *M. truncatula RCT1* (**C**); and flax *L6* (**D**). Exons are shown as boxes. The cryptic introns are indicated by vertically hatched boxes in the exons, and horizontally hatched boxes for cryptic exons in introns. The spliced and retained introns are shown as angled and straight lines, respectively. RT, regular transcript encoding the full-length protein product; AT, alternative transcript encoding an aberrant protein product; E, exon; I, intron.

The *Arabidopsis RPS4* gene confers resistance to *Pseudomonas syringae* pv. *tomato* strain DC3000 (DC3000) expressing *AvrRps4*. AS produces six transcript isoforms of *RPS4* via retention of intron 2 and/or intron 3, and splicing of a cryptic intron in exon 3 (Figure 1A) [28]. Due to premature stop codons introduced by frame shifts, the alternatively spliced isoforms encode no or fewer LRR repeats. Experiments involving stable transformation of *RPS4* genomic constructs lacking intron 2 and/or intron 3, under the control of the *RPS4* promoter, showed that deletion of a single intron was sufficient to abolish *RPS4* function, even though splicing of remaining intron was unaffected and the normally spliced transcript was also expressed [28]. Therefore, resistance to DC3000 requires AS of *RPS4*.

A role for these alternatively spliced isoforms as regulatory RNAs remains possible, but there is evidence that they encode truncated proteins that regulate the activity of full-length RPS4. An artificial combination of normal and alternatively spliced isoforms only partially restored *RPS4*-mediated resistance [28]. The molar ratio of *RPS4* transcript isoforms in that experiment was altered compared to those naturally occurring, suggesting that the ratio is of functional importance. The abundance of the various AS isoforms of *RPS4*, particularly the isoform retaining intron 3 (*RPS4_AT4_*), is under dynamic regulation in response to AvrRps4. Whereas the full-length transcript including all exons is the predominant splicing product in uninoculated leaves, pathogen inoculation induces a rapid, >100-fold increase of *RPS4_AT4_* [29]. The truncated proteins encoded by *RPS4* variants were detected in transient expression assays, confirming that the aberrant transcripts are functional.

#### 3.1.2. Tobacco *N*

Tobacco *N* specifically recognizes a 50-kDa helicase protein (p50) of tobacco mosaic virus (TMV), and the *N* gene is alternatively spliced [73,82]. In addition to the major isoform (*N_RT_*), an alternative isoform (*N_AT_*) is generated via AS of a hidden exon containing a stop codon within intron 3, which yields a putative product lacking 13 of 14 LRR repeats (Figure 1B). Similar to *RPS4*, a dynamic abundance ratio of *N_RT_* to *N_AT_* is also observed during TMV infection [30]. Although *N_RT_* is predominant before infection, *N_AT_* is the more abundant isoform 6 h after TMV inoculation, and the original isoform ratio reappears 9 h after inoculation. Perturbing the ratio of *N_RT_* to *N_AT_* resulted in compromised TMV resistance. The boost in *N_AT_* production may result from a signaling cascade induced by interaction between *N_RT_* and p50. Because the accumulation of spliced variants occurs rapidly, the induced AS may regulate N function via feedback inhibition. Tobacco transformants expressing only *N_RT_* displayed incomplete resistance manifested by delayed HR, which suggests that *N_AT_* is required for full *N*-mediated resistance [30]. However, *N_AT_* expressed alone was not sufficient for TMV-dependent HR.

#### 3.1.3. *M. truncatula RCT1*

*RCT1* confers resistance against multiple races of *Colletotrichum trifolii*, a hemi-biotrophic fungal pathogen that causes anthracnose disease in Medicago [81]. AS of *RCT1* results from the retention of intron 4, instead of intron 2 and/or intron 3 as in *N* and *RPS4* (Figure 1C). The alternative isoform (*RCT1_AT_*) is predicted to encode a truncated protein consisting of the entire TIR, NBS, and LRR domains, but lacking the *C*-terminal domain of the normal RCT1 protein (RCT1_RT_). *RCT1*-mediated resistance requires *RCT1_AT_* and *RCT1_RT_*, as transformants containing only *RCT1_AT_* or *RCT1_RT_* showed no anthracnose resistance [31]. Though the expression of *RCT1* transcripts was stable and constitutive, and unaffected by pathogen infection, a certain expression threshold for *RCT1_AT_* seemed to be essential for effective resistance.

#### 3.1.4. Flax *L6* and Tomato *Bs4*

In contrast to *RPS4*, *N* and *RCT1*, alternatively spliced transcripts of flax *L6* and tomato *BS4* are not required for full resistance to the corresponding pathogens. For example, transgenic plants carrying an intronless *L6* (*L6_RT_*) exhibited complete rust resistance, similar to plants carrying the wild-type *L6* (Figure 1D) [74]. L6 triggers flax rust resistance by direct interaction with its cognate effector *AvrL567*. The flax rust resistance gene *M*, which is homologous to *L*, is also alternatively spliced; therefore, it is possible that AS of the *M* locus could functionally substitute for AS of *L6*. This hypothesis was supported by *trans*-complementation of the *Rx* gene. *Rx* is a CC-NBS-LRR gene that confers resistance to potato virus X (PVX) elicited by a coat protein. The CC domains in Rx share 96% similarity with those of Gpa2, which is required for resistance to potato nematode [83,84]. The function of an NBS-LRR derivative of *Rx* (*Rx NBS-LRR*) lacking the CC domain can be complemented by *Gpa2* to induce coat protein-dependent HR [85]. Such *trans*-complementation appears to require high identity between the R proteins, because pepper *Bs2* (*Bacterial spot resistance gene 2*), which is homologous to *Rx*, failed to functionally complement an *Rx NBS-LRR* derivative.

Transient co-expression of *L6_RT_* and *AvrL6* in tobacco gives rise to apparent HR, which argues against any interference by the *M* locus [86]. Likewise, transient expression of intronless *Bs4* revealed that the normal Bs4 protein alone could mediate AvrBs4 recognition, which suggests that AS of *Bs4* is functionally dispensable [79]. Whereas such transient expression assays have served well for isolation of *R* genes [83], whether this system can reliably be used to analyze functional roles for AS of *R* genes remains to be established. It is possible that the observed HR could be due to partial resistance conferred by an endogenous full-length R protein, such as tobacco *N*. Recent analysis of truncated *R* genes containing TIR-NBS only (TN) in *Arabidopsis* showed that chlorosis was induced by transient overexpression of *TN* genes [87]. The alternative *L6* and *Bs4* isoforms were not tested in transient assays; therefore, these transient expression experiments may not fully reflect the physiological roles of AS in the process.

A stunted phenotype caused by constitutive defense responses was observed in transgenic tobacco carrying an *L6* genomic construct, as well as in transgenic tobacco plants in which *L6_RT_* was under the control of the 35S promoter [88]. This evidence is suggestive that AS is irrelevant to *L6*-mediated resistance, with dwarfism serving as a reporter for activation of defense responses. However, the lack of tobacco transformants expressing *L6_RT_* from its native promoter precludes firm conclusions about this. Structural and functional analysis demonstrated that the TIR domain alone is necessary and sufficient for *L6* immune signaling [89]. More interestingly, with only one exception (L10-A), tobacco plants transformed with a genomic construct of *L10* grew normally [88]. Further analysis revealed that the stunted phenotype of L10-A is associated with the presence of an additional truncated *L10* transcript resulting from an aberrant T-DNA integration [88]. This truncated transcript is predicted to encode a protein containing the TIR and 39 amino acids of the NBS domain of L10. These findings point to the possibility that the functional significance of AS in *L6* has been undervalued.

### 3.2. AS of CC-NBS-LRR Genes

AS has been identified in many CC-NBS-LRR *R* genes, including *LR10* and *Sr35* in wheat [90,91], *Mla* in barley [92,93], *Pi-ta* and *RGA5* in rice [94,95], and *JA1tr* in common bean [96], but the functional importance of this post-transcriptional modification for full disease resistance is largely unknown. Only the alternative transcripts of *RGA5* have been functionally characterized in a robust system [95].

Rice blast R protein RGA5 was found to cooperate with RGA4 in recognizing two sequence-unrelated effectors, Avr-pia and Avr1-CO39, through direct binding. Two transcript isoforms are generated by AS of the third of the three introns in the coding region of *RGA5* [97]. As in the case of *M. truncatula RCT1*, protein products of both the intronless, fully-spliced transcript (*RGA5_RT_*) and the AS version (*RGA5_AT_*) share the CC, NBS, and LRR domains, and differ only in the *C*-terminal region, which is related to the copper binding protein ATX1 (RATX1) [95]. Transformants carrying *RGA5_AT_* are fully susceptible to *Avr-pia*- and *Avr1-CO39*-expressing *Magnaporthe oryzae* strains. Furthermore, in conjunction with RGA4, RGA5_RT_ is necessary and sufficient to confer dual recognition specificity [95]. Yeast two-hybrid assays demonstrated that Avr-pia and Avr1-CO39 physically interact with the *C*-terminal RATX1 domain, which is present only in RGA5_RT_. The disruption of the RATX1 domain consequently renders RGA5_AT_ inactive. These findings highlight the importance of the non-LRR regions near the *C*-termini of R proteins, indicating that they may deserve more attention when exploring the functions of R proteins in disease resistance.

Another rice blast resistance gene, *Pi-ta*, confers resistance to strains of *M. oryzae* containing cognate avirulence gene *Avr-Pita*. A total of 12 distinct transcript isoforms were identified as resulting from AS and are predicted to encode 11 proteins. Some of these transcripts are constitutively expressed while others show differential expression upon blast infection [94]. Their regulatory roles in disease resistance remain unknown.

The barley powdery mildew resistance genes *Mla6* and *Mla13* have very similar gene structures, including the conservation of two introns in the 5'-UTR and two introns in the coding region, as well as a large intron in the 3'-UTR. Notably, both genes exhibit AS of the 5'-UTR, which contains three upstream ORFs (uORFs); AS is also predicted to cause variation of one amino acid in the coding region of *Mla13* [92]. The expression of *Mla13* transcripts is induced upon pathogen penetration, and a dynamic change in the relative abundance of transcript isoforms has been observed. Inactivation of uORF translation via mutagenesis suggests the uORFs in the 5'-UTR downregulate *Mla13* synthesis [98]. Hence, AS of uORFs may finely tune *Mla13* expression to achieve effective resistance while minimizing host cell damage. However, it remains unknown whether full resistance mediated by *Mla13* or *Mla6* requires AS of the uORFs.

## 4. Possible Mechanisms of AS-Mediated Regulation of Defense Response

In the cases where AS is necessary for disease resistance, transgenic plants containing only the full-length transcript do not display auto-immunity or lesion mimic phenotypes induced by increased R protein activity, suggesting that AS is not likely to negatively regulate the *R* gene function. By contrast, the absence of AS impairs *R* gene-mediated resistance, which is indicative of positive roles for AS in defense responses. R protein isoforms therefore possibly function by suppressing the negative regulation of immunity activation, or by directly engaging in effector-trigged signaling, or by a combination of both.

### 4.1. Disruption of R Protein Autoinhibition

Whether an R protein is active or inactive is determined by the binding of ATP or ADP to the NBS domain [99]. Since constitutive activation of R proteins leads to lethal effects on plant growth, negative regulation of R protein activity is essential [100,101,102]. Intramolecular interactions between R-protein domains may function as a regulatory switch, and several mechanistic models have been proposed to describe this R protein self-regulation, such as the “Jack-knife” model [103]. These models are based largely on the *trans*-complementation of Rx CC-NBS and LRR domains [85]. From the crystal structures of the TIR and CC domains [89,104], Takken and Goverse proposed a model in which the NBS domain interacts with the *N*-terminal half of the LRRs, maintaining the R protein as inactive in a closed conformation before pathogen invasion [105]. An electrostatic interface that maintains the inactive conformation may be formed by interaction between the LRR and NBS domains. The *C*-terminal LRRs are exposed to serve as an antenna to detect charge changes induced by environmental perturbations. Since the TIR or CC domain can also interact with the NBS domain [85], the R protein is stabilized in a compact structure in the absence of pathogens. Studies on intramolecular interactions of Rx have provided evidence that the NBS domain alone is not sufficient for stable binding, but instead requires the CC domain. Notably, the CC domain could also interact with the NBS domain, unless *N*-terminal LRRs were bound to the NBS domain [44,85,106]. As such, the interaction of LRRs and NBS domains seems to cause conformational changes in the latter that facilitate NBS binding with CC domain. It has also been demonstrated that the ARC1 subdomain is necessary for binding of the Rx *N*-terminal LRR domain, while the ARC2 subdomain is required to maintain an autoinhibited state in the absence of elicitor, as well as for subsequent signaling [44]. Mutation in LRRs or conserved ACR2 motifs of the NBS domain leads to the autoactivation of Rx and RPS5 [44,85,107]. The majority of truncated R protein variants generated by AS are presumably unstable, due to the lack of LRR domain, and it is thus speculated that the aberrant R protein isoforms induced by pathogen inoculation could form intermolecular interactions with their regular protein products. This would disrupt the closed conformation stabilized by intermolecular interactions and free active R proteins.

In addition to the autoinhibition, R proteins are also subjected to negative regulation by *trans* factors [103]. RIN4, guarded by RPM1 and RPS2, is phosphorylated upon infection with *P. syringae* by AvrRpm1 and AvrB [108,109]. The *rin4* mutants cannot survive in the presence of wild-type RPM1 and RPS2, due to strong activation of defense responses independent of pathogen infection. However, the *rin4* defective phenotype is suppressed in the triple mutant *rin4 rps2 rpm1* [103]. It was deduced that interactions of RIN4 with RPM1 and RPS2 negatively regulate the activities of both of these R proteins. The down-regulation of R protein activity could also be achieved by limiting its accumulation to a steady level. SRFR1 (Suppressor of RPS4-RLD1) interacts with SNC1 to negatively regulate production of several R proteins, such as RPS2, RPS4 and RPS6 [110]. Likewise, the F-box protein CPR1 (Constitutive expresser of PR genes 1) controls the stability of R proteins through SKP1-Cullin1-F-box (SCF)-mediated protein degradation [111]. Loss-of-function *cpr1* mutants displayed higher expression of SNC1 and RPS2, as well as autoimmunity responses. Excess R protein isoforms produced via AS upon effector recognition may compete with full-length R protein to interact with negative regulators and decrease the relative abundance of these suppressors, thereby releasing active R protein [103]. This assumption is in line with observations that the overexpression of some *R* genes, including *Rx*, *RPS2*, and *RPM1*, leads to constitutive activation of resistance signaling. 

### 4.2. Function as Signaling Factors

Overexpression of the TIR or CC domain of some R proteins (e.g., RPS4, RPP1, MLA10, and L6) can induce HR in the absence of cognate effectors [89,104,112,113,114]. In addition to the TIR-NBS-LRR-encoding *R* genes, plants also contain short pseudo-*R* gene homologs (*TN* and *TX*) [115]. TN proteins contain the TIR and NBS domains, but lack the LRR domain, while TX proteins have only the TIR domain followed by a small and variable *C*-terminal domain. *Arabidopsis* contains 21 *TN* and 30 *TX* genes [116]. Transient and stable overexpression of some *TN* and *TX* genes induced necrosis in tobacco leaves and reduced disease symptoms in *P. syringae*-infected *Arabidopsis* plants, respectively [87]. This suggests that the truncated R proteins resulting from AS may also confer disease resistance with or without recognition specificity.

The crystal structures of the TIR domain of L6 and CC domain of MLA10 indicated that two activated R proteins form a homodimer at the CC or TIR domain to constitute a minimal functional unit [89,104]. In the presence of full-length R protein, the production of massive amounts of truncated proteins containing TIR or CC may serve as a rapid and energy-efficient mechanism to activate responses to pathogen infection. If so, the rapid increase of TIR or CC domain-dependent dimerization stimulated by AS of *R* genes might function to amplify the plant defense responses.

Protein function is associated with subcellular localization. It is possible that the alternative proteins generated by AS are localized to different compartments than the full-length R proteins, and numerous reports have demonstrated dynamic subcellular localization for R proteins such as RPS4 and N [42,117,118]. Distinct signaling pathways can be initiated by a single R protein in different subcellular localizations, and, thus, the coordinated trafficking of R proteins is required for the activation of full resistance [119]. RPS4 is detected in both the endomembrane and nucleus in healthy and diseased leaves, with RPS4 accumulation in the nucleus appearing to be necessary for AvrRPS4-trigged immunity [118]. AvrRPS4 also shows a nucleo-cytoplasmic distribution. Forcing AvrRPS4 to accumulate in cytoplasm through the *C*-terminal fusion of a nuclear export sequence led to moderate HR and partial suppression of bacterial growth. By contrast, sequestration of AvrRPS4 in the nucleus by fusion of nuclear localization sequence was sufficient for inhibition of bacterial growth, but cell death elicited by HR was abolished. HR signaling is therefore mediated by cytoplasmic RPS4-AvrRPS4 interaction, whereas the nuclear R-Avr interaction-induced resistance is not coupled to programmed cell death. This is in line with the findings that restriction of pathogen spread does not always correlate with HR [120,121,122]. However, because the construct used for examination of RPS4 subcellular localization consisted of its genomic sequence with an upstream fusion of the reporter gene under the control of the 35S promoter, any differential targeting of full-length RPS4 compared to truncated variants could not be distinguished [117]. It is likely that the truncated RPS4 proteins would accumulate in the endomembrane system, since their *C* termini lack a bipartite nuclear localization sequence, which is necessary for accumulation of full-length RPS4 in the nucleus. This could explain why only 6%–10% of RPS4 was observed in the nuclei. The distinct types of signaling triggered by nucleo-cytoplasmic distribution of R-Avr interaction may be coordinated by AS and differential localization of the resultant protein isoforms.

Tobacco *N* is predicted to be cytoplasmic because it does not carry a recognizable nuclear localization signal. Unexpectedly, it was found to be localized to both the cytoplasm and the nucleus, and nuclear localization is required for N function [117]. Different constructs, tagged with distinct fluorescence genes for different *N* transcript isoforms, are needed to test whether AS leads to diverse subcellular localizations for alternative N isoforms.

## 5. Regulation of AS of *R* Genes

AS dramatically increases the diversity of the transcriptome, and AS of *R* genes plays crucial roles in regulating plant defense responses; therefore, the mechanisms that regulate AS must be finely tuned to control the levels of different AS transcripts. Removal of introns within pre-mRNA in eukaryotes is catalyzed by the spliceosome, a highly dynamic and complex macromolecule comprising five (U1, U2, U4, U5, and U6) small ribonucleoproteins (snRNPs) and numerous RNA binding proteins (RBPs), such as serine/arginine-rich (SR) proteins and heterogeneous nuclear ribonucleoproteins (hnRNPs). The precise selection of intron/exons requires splicing factors to recognize four loosely conserved sequence features in pre-mRNA: (**1**) the 5' splicing site (SS) of GU paired with snRNP U1; (**2**) a branch point A for binding of splicing factor 1 at the 18 to 40 nucleotides upstream of the 3' SS; (**3**) the 3' SS of AG and (**4**) a poly-pyrimidine tract for recruitment of U2 auxiliary factor heterodimer [26,123]. It is noteworthy that a single intron may contain multiple sites for each of these four conserved sequence elements, adding more complexity in splicing site selection.

Differential selection of 5'- or 3'-SSs can be also affected by some short sequences of *cis*-elements in intronic and exonic region. According to the position and function, these *cis*-elements are grouped as exonic splicing enhancers, exonic splicing silencers, intronic splicing enhancers, and intronic splicing silencers. These splicing regulatory elements bind to trans-acting splicing factors, such as SR proteins and hnRNPs, playing critical roles in both constitutive and alternative splicing through either inducing or suppressing selection of nearby 5'- or 3'-splicing sites [124,125]. Interestingly, *SR* genes are also extensively alternatively spliced and AS of *SR* genes is affected by environmental stresses such as temperature, light and salt, which in turn induces splicing changes in the pre-mRNAs of other genes [126].

As mentioned above, screening for suppressors of the gain-of-function mutation *snc1* led to the identification of a set of *MOS* genes, some of which function in pre-mRNA processing. For example, *Arabidopsis* mutants carrying a loss-of-function mutation for *MOS4*, *MOS12*, or *MOS14* show altered splicing patterns for *SNC1* and *RPS4*, which indicate that those genes have regulatory roles in AS of *R* genes [62,71,72]. MOS4, required for both ETI and PTI, is a nuclear localized CC homologous to human BCA2 (Breast cancer-amplified sequence 2). Together with the Myb-transcription factor CDC5L (Cell divison cycle 5 like protein) and the WD-40 repeat PLRG1 (Pleiotropic regulator 1), BCA2 was isolated from humans as an important component of a multiprotein spliceosome complex that includes the E3 ubiquitin ligase Prp19 (Precursor RNA processing 19) [127,128]. Yeast two-hybrid and *in planta* assays confirmed that MOS4 interacted with the *Arabidopsis* homologs of CDC5L and PRLG1 (AtCDC5 and PRL1, respectively) to constitute a core structure for a spliceosome-associated complex termed the MOS4-associated complex (MAC) [62]. MAC3A and MAC3B, two functionally redundant homologs of Prp19, contribute to proper splicing of *SNC1*, though their effects on AS of *RPS4* have not been investigated [129]. Similarly, whether two other redundant homologs, MAC5A and MAC5B, function in *R* gene AS has not been tested [130]. However, given that its counterpart in human is RBM22, which interacts with U6 snRNP, it is possible that MAC5 participates in pre-mRNA splicing in plants.

*MOS12* encodes an SR protein homologous to human cyclin L [72]. Co-immunoprecipitation of MOS12 with MOS4 indicates that MOS12 is also associated with the MAC. The *mos12* mutant displays compromised *RPS4*-mediated resistance as well as an altered splicing pattern of *RPS4*, leading to a different abundance ratio of *RPS4* transcript isoforms. However, the splicing pattern of *RPS6* is normal in the *mos12* mutant, as is *RPS6*-mediated resistance. This suggests that in addition to MAC, more spliceosomal complexes with distinct splicing specificities probably exist in plants.

Impaired *SNC1*- and *RPS4*-mediated PTI and ETI was also observed in the loss-of-function mutant of *MOS14* [71]. In addition to distorted splicing patterns, the *mos14* mutants showed reduced expression of *SNC1* and *RPS4*. *MOS14* encodes a nuclear protein homologous to transportin-SR, which functions in nuclear trafficking of the SR protein. MOS14 interacts with four different SR proteins through its *C*-terminus, while the *N*-terminus interacts with a GTP-binding protein AtRAN1 (Ras-related nuclear protein 1) which functions in many processes, including nuclear transport of proteins. The nuclear localization of these four proteins was disrupted in *mos14* mutants, which consequently affects the splicing profiles for their targets. Defective splicing resulting from mislocalization of MOS14 cargos may cause the reduction in *SNC1* and *RPS4* expression [71].

## 6. Future Prospects

The functional importance of AS in plant disease resistance has become increasingly clear. However, despite the substantial progress that has been made in the past decade, AS research in plant immunity is still in its infancy. The AS events characterized to date in CC-NBS-LRR genes appear not to be required for disease resistance. Therefore, more research on CC-NBS-LRR genes will be needed to confirm whether AS plays other functional roles. Some truncated TIR-NBS-LRR proteins encoded by alternative transcripts are required for full *R*-gene mediated resistance, which raises an interesting question of how they engage in *R*-*Avr* interactions. Neither the “elicitor-receptor” nor the “guard hypothesis” models explain how truncated R proteins function in triggering plant defense responses. The cognate Avr proteins for L6, RPS4 and N, and even their host targets [117,119,131] have been identified, providing an opportunity to discover biological roles for the aberrant R protein variants. Research in this direction will likely provide insights into the functional mechanisms of truncated R proteins, as well as their dynamic subcellular localization, and interactions with Avr proteins and their targets, thus extending our understanding of gene-for-gene resistance in plants.

The alternative transcript isoforms may be subjected to nonsense-mediated decay (NMD) [86], which is widespread in eukaryotes and serves as a quality control mechanism [132]. NMD is coupled with AS to regulate the levels of functional mRNA transcripts through the specific degradation of alternatively spliced isoforms possessing a premature stop codon [133]. However, it remains unclear that how the potential NMD targets derived from AS of *R* genes escape being destroyed. Although its functional roles are not clear and few targets have been identified, NMD regulation in disease resistance has been documented. *nmd* mutants display stunted phenotypes and curled leaves that resemble those of mutants with constitutive activation of defense responses [134], and the majority of transcripts enriched in *nmd* mutants are associated with the pathogen response [134].

The AS transcripts of *R* genes such as *N* and *RPS4* are of low abundance in the absence of their corresponding effectors. It was first assumed that NMD activity was repressed during pathogen infection, resulting in the accumulation of alternative *R* gene transcripts [135], which is consistent with the weak NMD activity observed by RNA-seq analysis in *P. syringae*-infected *Arabidopsis* [15]. Alternatively, the *R* gene transcript isoforms possessing premature stop codons may be insensitive to NMD. Generally, mRNAs targeted for NMD have uORFs or a larger 3'-UTR region [136]. However, many transcripts displaying intron retention are not sensitive to NMD, although they appear to have the characteristics of transcripts affected by NMD. Whatever the mechanism, more evidence is required to clarify the molecular machinery that suppresses NMD of alternatively spliced transcripts of *R* genes.

The cloning of the *MOS* genes shed light onto spliceosomal regulation in the AS of *R* genes. However, the splicing factors and RNA-binding proteins responsible for pre-mRNA splicing of *R* genes other than *SNC1* and *RPS4* are completely unknown. One challenge is that mutations in these factors may cause inconspicuous phenotypes, because AS could be unnecessary for full disease resistance. In addition, even if defense signaling requires AS of an *R* gene, the mutant would likely grow normally in the absence of the pathogen expressing its cognate effector. We therefore have a long way to go before the full picture of regulation of *R* gene AS is revealed. Continuous advances in genomics, bioinformatics, transcriptomics, phenomics, and sequencing technologies, should facilitate our explorations of the complexity for generation and contribution of AS of *R* genes and elucidate new avenues to modify plant innate immunity.

## Abbreviations

ARC: apaf-1, R and ced-4
Avr: avirulence
BCA2: breast cancer-amplified sequence 2
Bs4: bacterial spot resistance gene 4
CC: coil-coiled
Mla: mildew A
CDC5L: cell divison cycle 5 like protein
CPR1: constitutive expresser of PR genes 1
DC3000: *P. syringae* pv *tomato* strain DC3000
*Dscam*: down syndrome cell adhesion molecule
EDS1: enhanced disease susceptibility 1
EF-Tu: elongation factor-Tu
ETI: effector-triggered immunity
hnRNPs: heterogeneous nuclear ribonucleoproteins
HR: hypersensitive response
HRT: HR to TCV
LRR: leucine-rich repeat region
MAC: MOS4-associated complex
MOS: modifier of *snc1*
NBS: nucleotide-binding site
NDR1: non-race-specific disease resistance 1
NMD: nonsense-mediated decay
NPR1: non-expresser of PR genes 1
p50: 50-kDa helicase protein
PAD4: phytoalexin deficient 4
PAMPs or MAMPs: pathogen (or microbe)-associated molecular patterns
PLRG1: pleiotropic regulator 1
Prp19: precursor RNA processing 19
PRRs: pattern recognition receptors
PTI: PAMP-triggered immunity
RAC1: resistance to *Albugo candida* isolated ACEM1
RAN1: RAS-related nuclear protein 1
RBPs: RNA binding proteins
RGA: R-gene analog
RPP: recognition of *Peronospora parasitica*
RPS: resistance to *P. Syringae*
SCF: skp1-cullin1-F-box
snc1: suppressor of *npr1-1*, constitutive 1
snRNPs: small ribonucleoproteins
SR: serine-arginine rich
SRFR1: suppressor of RPS4-RLD1
TCV: turnip crinkle virus
TIR: *Drosophila* Toll and mammalian Interleukin-1 receptor
TMV: tobacco mosaic virus
TN: truncated R proteins containing TIR-NBS only
TX: truncated R proteins containing TIR only
uORFs: upstream ORFs
